# Linezolid-Associated Thrombocytopenia: Assessment of Risk Factors in Patients without Hemato-Oncologic Diseases

**DOI:** 10.3390/jcm13082380

**Published:** 2024-04-19

**Authors:** Abrar K. Thabit, Arwa A. Alghamdi, Afnan K. Alsaeed, Nesereen M. Magbool, Yazed S. Alsowaida, Ahmad J. Mahrous, Alya Alruwaili, Ziyad K. Albakistani, Basem O. Albangali, Anas M. Alghumuy, Sara A. Youssef, Reem M. Alodayli, Masaad Saeed Almutairi

**Affiliations:** 1 Pharmacy Practice Department, Faculty of Pharmacy, King Abdulaziz University, Jeddah 22254-2265, Saudi Arabia; 2 Faculty of Pharmacy, King Abdulaziz University, Jeddah 22254-2265, Saudi Arabia; 3 Department of Clinical Pharmacy, College of Pharmacy, University of Ha’il, Hail 55473, Saudi Arabia; 4 Clinical Pharmacy Department, College of Pharmacy, Umm Al Qura University, Makkah 21955, Saudi Arabia; 5 Department of Pharmaceutical Care, King Fahad Medical City, Riyadh 12231, Saudi Arabia; 6 College of Pharmacy, Umm Al Qura University, Makkah 21955, Saudi Arabia; 7 Department of Pharmaceutical Care, Saudi German Hospital, Hail 55481, Saudi Arabia; 8 Department of Pharmacy Practice, College of Pharmacy, Qassim University, Qassim 51452, Saudi Arabia; mas.almutairi@qu.edu.sa

**Keywords:** linezolid, thrombocytopenia, bacteremia, creatinine, creatinine clearance, risk factor, renal insufficiency

## Abstract

**Background**: Linezolid is used for Gram-positive bacterial infections. Thrombocytopenia is one of its main adverse effects resulting from myelosuppression. Several studies have assessed risk factors that may increase the risk of this adverse effect. However, most studies included patients with hemato-oncologic diseases, which may confound such assessments. This study aimed to investigate risk factors for linezolid-associated thrombocytopenia in patients without hemato-oncologic diseases. **Methods**: This was a multicenter retrospective case-control study of adult patients treated with linezolid twice daily for ≥3 days. Patients with hemato-oncologic diseases, active dengue fever, active COVID-19, baseline platelet count <100 × 10^3^/mm^3^, concurrent therapy with trimethoprim/sulfamethoxazole or valproic acid, and a recent platelet transfusion within 7 days were excluded. Thrombocytopenia was defined as a drop in platelet count below 100 × 10^3^/mm^3^. **Results**: Out of 158 evaluated patients, 33 developed thrombocytopenia, indicating an incidence rate of 20.9%. Of all the risk factors assessed, creatinine clearance of <60 mL/min and bacteremia/infective endocarditis were significantly associated with linezolid-associated thrombocytopenia (adjusted odds ratios, 3.25 and 5.95; 95% CI 1.12–9.45 and 1.23–28.66; *p* = 0.031 and 0.026, respectively). End of therapy platelet counts were significantly lower in the cases than in the controls (79 vs. 243 × 10^3^/mm^3^; *p* < 0.001). Similarly, the percentage of platelet count change was significantly different (−55.1% vs. −10.2%; *p* < 0.001). **Conclusions**: In our study, the incidence rate of linezolid-associated thrombocytopenia was 20.9%, and we found that patients with renal impairment and bacteremia may need close monitoring of platelet counts. Prospective studies are warranted to evaluate the potential need for renal dose adjustment.

## 1. Introduction

Linezolid is the first member of the oxazolidinone class of antibiotics [1]. It exhibits its activity by inhibiting the protein synthesis of Gram-positive cocci, including *Staphylococcus aureus* (both methicillin-resistant and susceptible), *Streptococci*, *Enterococci* (including vancomycin-resistant strains), and *Listeria monocytogenes* [2]. Common adverse effects associated with linezolid use are thrombocytopenia, peripheral and ocular neuropathy, anemia, and lactic acidosis [1]. Thrombocytopenia is a consequence of myelosuppression, usually occurring after long-term linezolid treatment [3]. In the literature, the incidence of thrombocytopenia varied between 16.7% and 60.5% [3,4,5,6,7,8]. Such adverse effects can lead to treatment discontinuation due to the increased risk of bleeding [9]. The mechanisms of linezolid-associated thrombocytopenia are not clearly identified.

A considerable body of literature has studied the incidence and risk factors of linezolid-associated thrombocytopenia. Most of these studies were limited to patients with hemato-oncologic diseases, which may confound the assessment of thrombocytopenia. Receiving linezolid therapy for more than 14 days, low pretreatment platelet counts, renal insufficiency, chronic liver disorder, respiratory tract infections, and low body weight have all been reported as potential risk factors in these patients [3,10,11,12,13]. Moreover, intensive care unit patients who developed thrombocytopenia after treatment with linezolid had remarkably higher mortality [14]. One study evaluated the incidence and clinical characteristics of linezolid-associated thrombocytopenia in 19 patients treated with linezolid. Overall, thrombocytopenia (platelet count < 100,000 platelets/mm^3^) was observed in 32% of patients who received linezolid for more than 10 days [5]. Other studies found that linezolid treatment duration, renal function, and hepatic function play a key role in linezolid-associated thrombocytopenia [6,8,11].

To date, only a few studies have investigated factors that may increase the risk of linezolid-associated thrombocytopenia in patients without hemato-oncologic diseases. These studies have established a variety of risk factors, such as linezolid therapy for ≥7 days, baseline platelet count < 150 × 10^3^/mm^3^, creatinine clearance (CrCl) < 30 mL/min, concurrent low-dose aspirin therapy, a higher daily per kg dose, and a high white blood cell (WBC) count [15,16]. Nonetheless, no studies have assessed the potential effects of artificial heart valve surgeries and blood vessel grafts, and only one study reported the risk of concomitant aspirin therapy. It is important to ensure the safe use of linezolid by preventing its adverse effects through early identification of risk factors that may increase the incidence of linezolid-associated thrombocytopenia. Therefore, this study aimed to add to the current body of evidence and investigate risk factors for linezolid-associated thrombocytopenia in patients without hemato-oncologic diseases who received linezolid.

## 2. Methods

### 2.1. Study Design and Definitions

This was a multicenter retrospective case-control study of patients receiving linezolid therapy in four tertiary hospitals during the period from January 2013 to December 2022.

The primary endpoint was thrombocytopenia, which was defined as a decrease in platelet count to <100 × 10^3^/mm^3^ (i.e., less than approximately 75% of the lower limit of normal of 150 × 10^3^/mm^3^) [17]. Cases were defined as patients who developed thrombocytopenia during therapy with linezolid and up to 10 days post therapy, whereas controls were patients who did not develop thrombocytopenia despite exposure to linezolid. Several patient- and treatment-related factors were assessed for the potential augmentation of linezolid-associated thrombocytopenia.

### 2.2. Inclusion and Exclusion Criteria for Patients

Included patients were hospitalized adults (≥18 years) who received oral or intravenous (IV) linezolid at a dose of 1200 mg daily (600 mg twice daily) for at least 72 h. Patients having hemato-oncologic disease (including iron deficiency anemia, hemophilia, sickle cell disease, thalassemia, bone marrow transplant, leukemia, and lymphoma as well as cancer of other organs), active dengue fever, active coronavirus infection 2019 (COVID-19), baseline platelet count < 100 × 10^3^/mm^3^, concomitant therapy with trimethoprim/sulfamethoxazole and valproic acid, received platelet transfusion within 7 days prior to linezolid therapy, had no baseline or end of therapy laboratory platelet data (at least within 3 days of each time point) were excluded from the study. Patients with active dengue fever and COVID-19 were excluded due to the high rate of thrombocytopenia associated with these conditions [18,19].

### 2.3. Ethical Approval

The study protocol was approved by the Biomedical Ethics Committee of the Faculty of Medicine (reference no. 464-20), which waived the requirement for patient consent given the retrospective nature of the study.

### 2.4. Statistical Analysis

Categorical data were presented as numbers and percentages and compared using the chi-square test, whereas continuous data were presented as the median [interquartile range, IQR] and compared using the Mann–Whitney test. The Shapiro–Wilk test of normality showed the lack of a normal distribution for all continuous variables. Multivariable regression was performed for variables with a *p* < 0.1 in the univariate analysis to generate adjusted odds ratios (AOR). The omnibus test of model coefficients was used to determine the significance of the model, whereas the Hosmer–Lemeshow test was used to establish the goodness of fit of the model. A log-rank test was performed to assess the effect of these factors on the platelet count trend over time. A *p* value of <0.05 was used to determine statistical significance. SPSS version 24.0 software (IBM, Corp., Armonk, NY, USA) was used for analysis.

### 2.5. Sample Size Calculation

Based on two previous similar studies on patients without hemato-oncologic diseases that found a thrombocytopenia rate of 29.2% and 42%, respectively, a total of 145 patients were needed to meet a study power of 95% considering a difference in the incidence of thrombocytopenia (i.e., effect size) of 30% (rounded down from a mean of 35.6% to maximize the sample size) and an α-error probability of 5% [15]. This effect size was selected based on this study since it was the only study that was found in the literature that excluded patients who had hemato-oncologic diseases.

## 3. Results

### 3.1. Baseline Characteristics

A total of 158 patients met the eligibility criteria and were included in the study. Table 1 lists the characteristics of patients. The median age of the whole cohort was 62 years, with the majority being males (57.6%). Median [IQR] durations of linezolid therapy were 8 [6.5–10.5] vs. 7 [5–9.5] days (*p* = 0.344) in the cases and control groups, respectively. More than half of the patients were in the intensive care unit (58.2%), and many (70.3%) received linezolid intravenously. Notably, more than half of the patients (61.1%) had a creatinine clearance (CrCl) < 60 mL/min, with the majority being from the cases group (81.8% vs. 55.6%; *p* = 0.006).

### 3.2. Platelet Outcomes

**Figure 1:**
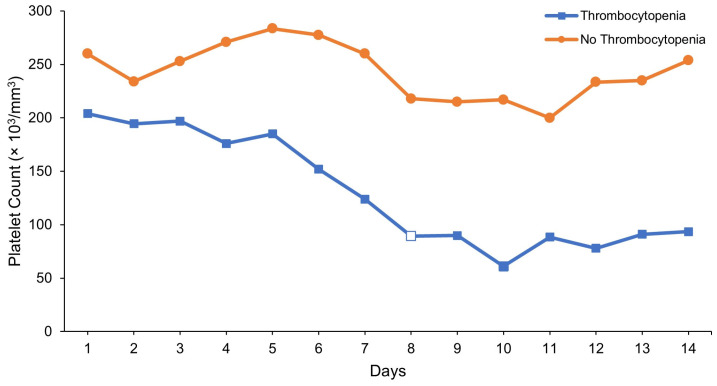
Trend of platelet counts over 14 days in the cases (thrombocytopenia; *n* = 33) and the controls (no thrombocytopenia; *n* = 125). The white square indicates the median duration of thrombocytopenia (platelet count < 100 × 10^3^/mm^3^).

Table 2 lists the platelet count outcomes in both groups. Overall, thrombocytopenia occurred in 33 patients, indicating an incidence rate of 20.9%. The median duration of thrombocytopenia was 8 days post-linezolid initiation. Figure 1 shows the trend of platelet counts over 14 days in the cases and the controls.

### 3.3. Factors Associated with Thrombocytopenia

In the univariate analysis, several factors were significantly associated with linezolid-associated thrombocytopenia, including low CrCl, previous vancomycin use, bacteremia, leukocytosis, and low hemoglobin (Table 3). However, only low CrCl (AOR, 3.25; 95% CI, 1.12–9.45, *p* = 0.031), bacteremia with or without infective endocarditis as linezolid indication (AOR, 5.95; 95% CI, 1.23–28.66, *p* = 0.026), and low baseline platelet count (AOR, 3.22; 95% CI, 1.01–10.26; *p* = 0.048) remained significantly associated with linezolid-associated thrombocytopenia in the multivariable regression analysis. Additionally, results of log-rank tests assessing the effect of all these factors on the platelet count trend over time showed no significant difference between patients who had the factors and those who did not.

## 4. Discussion

Linezolid is an important antibiotic for several infections, and it is widely used in clinical practice. In our study, we evaluated the incidence rate of linezolid-associated thrombocytopenia and identified the risk factors that may potentially augment this risk. In our study, 33 of 158 patients (20.9%) developed thrombocytopenia with linezolid therapy. We found that impaired kidney function, the presence of bacteremia with or without infective endocarditis, and a low baseline platelet count were significantly associated with thrombocytopenia in patients receiving linezolid for therapy.

The incidence rate of thrombocytopenia with linezolid therapy reported in the package insert is 2.4% [20]. Several studies have evaluated factors that may increase the risk of linezolid-associated thrombocytopenia. Choi et al. evaluated risk factors for linezolid-associated thrombocytopenia in 264 patients without a history of hemato-oncologic conditions [15]. They found a thrombocytopenia incidence of 29.2%, where a duration of linezolid therapy of 7 days or longer was significantly associated with thrombocytopenia (AORs were 7.25, 19.51, and 28.80; 95% CI, 1.92–27.38, 4.76–79.95, and 6.48–127.92 for 7–13 days, 14–20 days, and ≥21 days, respectively; *p* < 0.01 for all durations). Furthermore, they found that a baseline CrCl of <30 mL/min (AOR, 4.19; 95% CI, 1.59–11.06; *p* = 0.004), concomitant low-dose aspirin therapy (≤160 mg/day) (AOR 2.99; 95% CI, 1.26–7.08; *p* = 0.013), and a baseline platelet count of <150 × 10^3^/mm^3^ (AOR, 5.08; 95% CI, 2.06–12.55; *p* < 0.001) were significantly associated with thrombocytopenia associated with linezolid therapy. While our study also showed a correlation between low CrCl and low baseline platelet counts and the increased risk of linezolid-associated thrombocytopenia, our findings did not suggest a correlation with the use of any antiplatelet drug used by our patient population. Another retrospective study by Han, et al. evaluated 320 patients on linezolid therapy, where they found that thrombocytopenia (platelet count < 100 × 10^3^/mm^3^) was significantly associated with a low baseline platelet count (OR, 0.99; 95% CI, 0.98–0.99; *p* < 0.001), a long duration of linezolid therapy (OR, 3.46; 95% CI, 1.80–6.68; *p* < 0.001 for 7–13 days and OR, 2.51; 95% CI, 1.05–6.00; *p* = 0.039 for 14 days or longer), and shock (OR, 2.09; 95% CI, 1.13–3.88; *p* = 0.019) [6]. However, only a long duration of linezolid therapy (OR, 3.77; 95% CI, 2.21–6.41; *p* < 0.001 for 7–13 days and OR, 2.62; 95% CI, 1.26–5.43; *p* = 0.010 for 14 days or longer) and shock (OR, 1.91; 95% CI, 1.13–3.21; *p* = 0.015) were associated with a ≥25% drop in platelet count from baseline. The impact of a low baseline platelet count on linezolid-associated thrombocytopenia was also seen in our study; though, the duration of therapy (≤7 days vs. >7 days) did not impact such an incidence. Interestingly, low baseline platelet counts of ≤200 × 10^3^/mm^3^ also significantly increased the risk of linezolid-associated thrombocytopenia in a small study of 66 patients (AOR, 5.66; 95% CI, 1.15–27.9; *p* = 0.045) [7]. Similar to our findings, the study also found that a baseline CrCl < 60 mL/min and a baseline serum creatinine of >1.5 mg/dL were associated with a higher risk of linezolid-associated thrombocytopenia (AOR, 9.41; 95% CI, 1.09–80.54; *p* = 0.043 and AOR, 4.57; 95% CI, 1.26–16.5; *p* = 0.035, respectively). A therapy duration of >10 days was also seen as a factor associated with linezolid-associated thrombocytopenia in a case series of 19 patients, where 6 patients (32%) developed this adverse effect [5].

A potential explanation behind the increased risk of linezolid-associated thrombocytopenia in patients with renal impairment is that linezolid and its two major metabolites (aminoethoxyacetic acid metabolite and hydroxyethyl glycine) were found to accumulate in such patients, particularly in patients with end-stage renal disease [21]. However, the mechanism of thrombocytopenia associated with linezolid, including the potential effects of its two metabolites, is not yet fully understood.

In our study, we noticed that patients with bacteremia with or without infective endocarditis have an increased risk of thrombocytopenia in patients receiving linezolid for therapy (AOR, 5.95; 95% CI, 1.23–28.66; *p* = 0.026). This could be attributed to the bacterial effect on platelets. For instance, *S. aureus* is known to activate and aggregate platelets, as observed in previous in vitro studies [22,23]. Clinically, multiple studies found thrombocytopenia rates of 20–47.6% in patients with bacteremia due to various pathogens [24,25,26].

The median time to thrombocytopenia in our study was 8 days (IQR, 5.5–11). Several studies have addressed the time to thrombocytopenia after the initiation of linezolid. Giunio-Zorkin et al. assessed the new onset of thrombocytopenia caused by linezolid in a small study of 18 patients [27]. They found that the first thrombocytopenic values occurred within 14 days in 9 of 18 (50%) of the cases, resulting in a discontinuation rate of 61% (11/18), while one patient received a platelet transfusion. While this study did not exclude cancer patients as in the case of our study, they excluded patients with hematological disorders associated with platelet reduction, as well as patients who received bone marrow suppressive therapy within two weeks before linezolid initiation. Although the incidence of thrombocytopenia occurred later than in our study (8 days), newer and larger studies found that the incidence of thrombocytopenia occurred sooner, as in the study by Choi, et al., who found it happening after a median of 11.2 days [15]. In a small prospective study of 31 patients, Nukui, et al. found that the median time to thrombocytopenia was 11 days after the initiation of linezolid treatment, which occurred in 17 patients, indicating an incidence rate of 56.7% [28]. In their study, the authors measured the plasma concentration of linezolid and assessed the kidney function of their patients. They found that patients who developed thrombocytopenia had significantly higher trough concentrations of linezolid, particularly in patients with baseline CrCl < 60 mL/min. Regardless of kidney function, median linezolid trough concentrations in patients who developed thrombocytopenia vs. those who did not develop it were 13.4 mg/L vs. 4.3 mg/L on day 3, 15.3 mg/L vs. 3.8 mg/L, and 15.2 mg/L vs. 5 mg/L on days 3, 7, and 14, respectively (*p* < 0.0001). When divided based on CrCl value, median linezolid trough concentrations were 14.7 mg/L vs. 4.8 mg/L on day 3, 16.3 mg/L vs. 4.9 mg/L, and 13.6 mg/L vs. 7.3 mg/L on days 3, 7, and 14, respectively, in patients with CrCl < 60 mL/min vs. those with higher CrCl values (*p* < 0.0001). As such, the authors found that the strongest predictors of linezolid-associated thrombocytopenia were a CrCl < 60 mL/min (OR, 39; 95% CI, 3.8–399.8; *p* = 0.0002) and a linezolid trough concentration > 7.5 mg/L on day 3 (OR, 90; 95% CI, 7.3–1115.9; *p* < 0.0001). Findings from this study coincide with our findings that renal impairment at baseline can be a predictor of thrombocytopenia with linezolid, which warrants closer monitoring of platelet counts in patients with such a condition. When compared with its first alternative, vancomycin, linezolid was found to be associated with thrombocytopenia four times more than vancomycin (OR 4.39; 95% CI 2.38–8.08), as reported in a retrospective study of 453 patients by Al-Harbi, et al. [29]. In this study, the onset of severe thrombocytopenia (platelet count < 50 × 10^3^/mm^3^) occurred within an average of 14 ± 9.3 days of linezolid initiation. Although all these studies had a later onset of thrombocytopenia than in our study (8 days), our findings indicate that platelet monitoring should be started within a few days after treatment initiation.

Studies conducted on patients with hemato-oncologic diseases found that the most common factors associated with increased risk of linezolid-associated thrombocytopenia were poor kidney function (CrCl < 50 mL/min) (*p* < 0.001), duration of linezolid therapy of ≥14 days (*p* < 0.001), concomitant use of fluoroquinolones (*p* = 0.009), and respiratory tract infections (*p* = 0.03) [3]. In patients with a CrCl of < 50 mL/min, the time to the onset of thrombocytopenia was significantly shorter than in patients with a CrCl of >50 mL/min (*p* = 0.04) [3]. In our study, we excluded patients with hemato-oncologic conditions because these conditions are significantly associated with thrombocytopenia even without the presence of linezolid, which may confound our results. We also excluded patients with active dengue fever (which has been reported in our country) given the high rate of reported thrombocytopenia, reaching 90.1% [30]. Although one study found that parenteral administration of linezolid resulted in a higher rate of thrombocytopenia (*p* = 0.04) [3], no such difference was found in our study between oral and parenteral formulations, perhaps due to the high oral bioavailability of linezolid, which can reach approximately 100% [20].

In our study, we excluded patients with COVID-19 to match the inclusion criteria of a previous similar study by Han, et al. [6]. This was also because COVID-19 has been linked to thrombocytopenia of varying degrees, as reported in previous studies, case reports, and case series [19,31,32,33,34,35,36,37,38]. Thus, we wanted to exclude this condition as a potential confounding factor. Moreover, since our study spanned from before COVID-19 (2013–2019) until 3 years during/after COVID-19 (2020–2022), patients with COVID-19 would have been only included from the last 3 years of the study, which may have created an imbalance in the distribution of patients across the study period. Nonetheless, a study by Alkhalifa, et al. found that exposure to linezolid increased the likelihood of thrombocytopenia in patients with COVID-19 (chi-square *p* < 0.05); though, the authors did not mention the number of patients who received linezolid among the 256 included patients [39]. Therefore, it is suggested to assess the impact of a current or previous episode of COVID-19 on the incidence of thrombocytopenia in patients receiving linezolid.

Dengue fever is a viral infectious disease that impacts total blood cellular counts, including platelets. The World Health Organization guidelines included thrombocytopenia as one of the manifestations that correlate with the clinical severity of the disease [18]. A study by Castilho, et al. found a thrombocytopenia incidence rate of 40.3% in patients with laboratory-confirmed dengue fever [40]. The study did not assess the potential impact of concomitant drugs, including linezolid, on such incidence. However, the already high rate of thrombocytopenia associated with dengue fever may have made it a significant confounding factor if patients with the disease were to be included in the current study; therefore, we opted to exclude them. Nevertheless, evaluating the impact of both factors combined (i.e., linezolid and dengue fever) on the incidence of thrombocytopenia is worthy of investigation in a future study.

This study was perhaps limited by its sample size, despite the involvement of four medical centers. This can be attributed to the fact that linezolid was not available in our country until 2012 and that it is usually reserved for cases where other cheaper therapies (for example, vancomycin or trimethoprim/sulfamethoxazole) cannot be used, such as in cases of vancomycin resistance or drug-resistant tuberculosis. We also excluded several patients who matched the exclusion criteria to eliminate a potential confounding effect. As such, and given the observed incidence of linezolid-associated thrombocytopenia in our study, patients were included in a 1:3 ratio (i.e., one case for every three controls). Despite being a multicenter study, the limited sample size may limit the generalizability of the findings, especially for patients with conditions that were excluded from the study. Additionally, the retrospective design of the study may have introduced selection bias. Furthermore, to overcome information bias, we excluded patients with insufficient platelet data in their electronic medical records.

## 5. Conclusions

Linezolid represents an important therapeutic option for many kinds of infections, particularly those caused by drug-resistant Gram-positive bacteria or tuberculosis. The incidence rate of linezolid-associated thrombocytopenia in our study was 20.9%. Clinicians should exercise caution when using it in patients with impaired kidney function, bacteremia and/or infective endocarditis, and a low pre-treatment platelet count by close and frequent monitoring of the platelet count and the potential risk of bleeding. Alternatively, seeking another antibiotic to which the pathogen is susceptible may be more prudent for patients at risk while weighing the benefit vs. risk ratio. Future research is needed to address gaps in the knowledge of the mechanism behind such an adverse effect of linezolid and its metabolites, as well as the potential compounding effect of the excluded conditions, such as dengue fever and COVID-19.

## Figures and Tables

**Table 1:** Baseline characteristics of the patients.

Characteristic	All Patients (*n* = 158)	Cases (*n* = 33)	Controls (*n* = 125)	*p* Value
Age, years	62 [47.5–74]	62 [57–76]	62 [42–73]	0.165
Sex, male	91 (57.6)	20 (60.6)	71 (56.8)	0.694
Body mass index (*n* = 156), kg/m^2^	26 [23–30]	26 [24–31]	26 [23–30]	0.639
Location				0.479
Inpatient medical ward	66 (41.8)	12 (36.4)	54 (43.2)	
Intensive care unit	92 (58.2)	21 (63.6)	71 (56.8)	
Route of linezolid administration				0.437
Oral	47 (29.7)	8 (24.2)	39 (31.2)	
Intravenous	111 (70.3)	25 (75.8)	86 (68.8)	
Duration of therapy, days	7.5 [5–10]	8 [6.5–10.5]	7 [5–9.5]	0.344
Duration of therapy				0.328
≤7 days	79 (50)	14 (42.4)	65 (52)	
>7 days	79 (50)	19 (57.6)	60 (48)	
History of cardiovascular disease	89 (56.3)	23 (69.7)	66 (52.8)	0.082
Having more than one cardiovascular disease	45 (50.6)	13 (56.5)	32 (48.5)	0.507
History of cardiovascular conditions				0.321
Cerebrovascular accident	40 (25.3)	8 (24.2)	32 (25.6)	
Myocardial infarction	36 (22.8)	10 (30.3)	26 (20.8)	
Arrhythmia	22 (13.9)	7 (21.2)	15 (12)	
Heart failure	16 (10.1)	5 (15.2)	11 (8.8)	
Deep venous thrombosis	12 (7.6)	4 (12.1)	8 (6.4)	
Angina	9 (5.7)	3 (9.1)	6 (4.8)	
Cardiomyopathy	4 (2.5)	1 (3)	3 (2.4)	
Pulmonary embolism	4 (2.5)	1 (3)	3 (2.4)	
Valvular heart disease	2 (1.3)	1 (3)	1 (0.8)	
Rheumatic heart disease	2 (1.3)	1 (3)	1 (0.8)	
History of cardiovascular surgery	13 (9.2)	3 (10)	10 (9)	0.868
Type of cardiovascular surgery				0.276
CABG	9 (5.7)	1 (3)	8 (6.4)	
Heart valve	4 (2.5)	2 (6.1)	2 (1.6)	
Creatinine clearance				0.006
≥60 mL/min	61 (38.9)	6 (18.2)	55 (44.4)	
<60 mL/min	96 (61.1)	27 (81.8)	69 (55.6)	
Indication for linezolid				
Pneumonia	53 (33.5)	5 (15.2)	48 (38.4)	0.012
Empiric	47 (39.7)	13 (39.4)	34 (27.2)	0.173
Soft and skin tissue infections	16 (10.1)	2 (6.1)	14 (11.2)	0.384
Urinary tract infection	14 (8.9)	4 (12.1)	10 (8)	0.459
Bacteremia/infective endocarditis	11 (7)	6 (18.2)	5 (4)	0.004
Intra-abdominal infection	7 (4.4)	1 (3)	6 (4.8)	0.660
Tuberculosis	5 (3.2)	2 (6.1)	3 (2.4)	0.285
Central nervous system	3 (1.9)	0 (0)	3 (2.4)	0.369
Bone and joint infection	2 (1.3)	0 (0)	2 (1.6)	0.465
Use of antiplatelet drugs	60 (38)	16 (48.5)	44 (35.2)	0.162
Antiplatelet drug used *				0.244
Aspirin	42 (26.6)	10 (30.3)	12 (9.6)	
Clopidogrel	39 (24.7)	12 (36.4)	27 (21.6)	
Ticlopidine	1 (0.6)	0 (0)	1 (1.6)	
Previous vancomycin use within 14 days	46 (29.1)	14 (42.4)	32 (25.6)	0.058
Baseline blood laboratory data				
Platelets < 150 × 10^3^/mm^3^	19 (12)	8 (24.2)	11 (8.8)	0.015
Low hemoglobin **	118 (77.1)	29 (93.5)	89 (73)	0.015
Leukocytosis	93 (58.9)	14 (42.4)	73 (63.2)	0.031

Data are presented as *n* (%) or median [interquartile range]; CABG, coronary artery bypass graft; * 18 patients received both aspirin and clopidogrel; ** The lower limit of normal is 14 g/dL for males and 12 g/dL for females.

**Table 2:** Outcomes of platelet count in patients who developed and did not develop thrombocytopenia.

Outcome	All Patients (*n* = 158)	Cases (*n* = 33)	Controls (*n* = 125)	*p* Value
Time to thrombocytopenia, days	N/A	8 [5.5–11]	N/A	N/A
EOT platelet count, ×10^3^/mm^3^	213 [132–298]	79 [63.5–114]	243 [168.5–328.5]	<0.001
EOT change in platelet count, %	−15.4 [−46.6–17.2]	−55.1 [−76.2–−37.85]	−10.2 [−30–21.3]	<0.001

Data are presented as median [interquartile range]; EOT, end of therapy; N/A, not applicable.

**Table 3:** Factors associated with thrombocytopenia in patients on linezolid therapy.

Factor	Univariate Analysis			Multivariable Analysis		
	OR	95% CI	*p* Value	AOR	95% CI	*p* Value
Previous vancomycin use	0.47	0.21–1.04	0.058	1.07	0.40–2.88	0.893
History of cardiovascular disease	2.06	0.90–4.67	0.082	1.52	0.59–3.88	0.386
Bacteremia or infective endocarditis	0.19	0.05–0.66	0.004	5.95	1.23–28.66	0.026
Pneumonia	3.50	1.26–9.62	0.012	0.39	0.13–1.21	0.104
CrCl < 60 mL/min	3.59	1.38–9.3	0.006	3.25	1.12–9.45	0.031
Baseline platelets < 150 × 10^3^/mm^3^	0.30	0.11–0.83	0.015	3.22	1.01–10.26	0.048
Leukocytosis	2.33	1.07–5.08	0.031	0.66	0.26–1.64	0.369
Low hemoglobin	0.19	0.04–0.82	0.015	3.30	0.64–17.01	0.154

AOR, adjusted odds ratio; CrCl, creatinine clearance; OR, odds ratio.

## Data Availability

The datasets generated and analyzed in the current study are available from the corresponding author at reasonable request.
